# Proposed Training to Meet Challenges of Large-Scale Data in Neuroscience

**DOI:** 10.3389/fninf.2016.00028

**Published:** 2016-07-18

**Authors:** William Grisham, Barbara Lom, Linda Lanyon, Raddy L. Ramos

**Affiliations:** ^1^Department of Psychology, University of CaliforniaLos Angeles, CA, USA; ^2^Biology Department, Davidson CollegeDavidson, NC, USA; ^3^International Neuroinformatics Coordinating FacilityStockholm, Sweden; ^4^Department of Biomedical Sciences, College of Osteopathic Medicine, New York Institute of TechnologyOld Westbury, NY, USA

**Keywords:** training programs, big data, teaching, pedagogy, workforce preparation, analyses skill sets

## Abstract

The scale of data being produced in neuroscience at present and in the future creates new and unheralded challenges, outstripping conventional ways of handling, considering, and analyzing data. As neuroinformatics enters into this big data era, a need for a highly trained and perhaps unique workforce is emerging. To determine the staffing needs created by the impending era of big data, a workshop (iNeuro Project) was convened November 13–14, 2014. Participants included data resource providers, bioinformatics/analytics trainers, computer scientists, library scientists, and neuroscience educators. These individuals provided perspectives on the challenges of big data, the preparation of a workforce to meet these challenges, and the present state of training programs. Participants discussed whether suitable training programs will need to be constructed from scratch or if existing programs can serve as models. Currently, most programs at the undergraduate and graduate levels are located in Europe—participants knew of none in the United States. The skill sets that training programs would need to provide as well as the curriculum necessary to teach them were also discussed. Consistent with *Vision and Change in Undergraduate Biology Education: A Call to Action*^1^, proposed curricula included authentic, hands-on research experiences. Further discussions revolved around the logistics and barriers to creating such programs. The full white paper, *iNeuro Project Workshop Report*, is available from iNeuro Project^2^.

## Introduction

Large-scale brain genomics has already made a significant impact on neuroscience (Insel et al., 2004), and big data have the potential to change the process of discovery in neuroscience as it has in mathematics, astronomy, and genetics (Nielsen, 2011). Although data in neuroscience is being produced at ever larger scales, the promise of this large-scale data can only be realized by having a workforce adequately trained to meet its challenges. The scale of data being considered by current initiatives creates challenges that outstrip conventional ways of handling, considering, and analyzing data (see *Priorities for Accelerating Neuroscience Research Through Enhanced Communication, Coordination, and Collaboration*^3^). Large-scale neuroscience projects emerging around the world—such as the European Commission Human Brain Project^4^ and the White House BRAIN Initiative^5^—demand big data neuroinformatics approaches. These approaches include multi-scale integration of the dynamic activity and structure of the brain, brain simulation, quantitative theory and modeling of brain function, neurotechnology and research infrastructure, neuromorphic computing, theoretical neuroscience, and large-scale brain activity maps (Insel et al., 2013).

Big data projects create significant computational challenges and require the development and deployment of new methods, algorithms, and tools for visualization, analysis, and data mining. Further, big data entails sharing and amalgamating data, which requires ontogenies, standards, and ontologies to link, describe, and maintain data in usable fashions. The field of neuroscience faces particular challenges due to the multi-scale nature of the data and the need to integrate across many sub-domains and species. Integration and indexing data across scales and complex data repositories is essential to identify meaningful patterns and to enable researchers to build efficiently upon prior work. At present, there are few programs worldwide that are geared toward properly training students to meet these challenges.

The next generation workforce will be dealing with this large-scale data and therefore needs the appropriate skills and knowledge to fulfill these roles. Key players such as the National Science Foundation (NSF) and International Neuroinformatics Coordinating Facility (INCF) see workforce development as an essential element in realizing the exciting potential of big data in neuroscience.

Inspired by the efforts and priorities of the European Commission Human Brain Project and the White House BRAIN Initiative, an NSF-sponsored workshop, called iNeuro Project, with more than 35 participants was convened in Arlington, VA, USA, November 13–14, 2014 to address the need for training in this aspect of neuroinformatics. This workshop brought together purveyors of large-scale data resources, individuals involved with bioinformatics training, library and information scientists, computer scientists, neuroscience educators, INCF and NSF officers, and other scientific collaborators focused on dealing with the human capital needs posed by large-scale data in neuroscience. Here we report on the discussions of this workshop both to inform the community and as a call to action to develop programs to educate and train the workforce needed to fulfill the potential of big data.

This workshop was designed to: (1) obtain a statement of the problem from various perspectives; (2) discern where we are now in terms of training this future workforce; (3) decide what desired skill sets will be needed in the future; (4) discern the curricular mix needed to impart the desired skill sets; (5) discuss whether existing training programs can serve as models for the desired training or whether these programs would have to be developed *de novo*; (6) discuss the strengths and weaknesses of those proposed training programs; and (7) plan for next steps. The full workshop report is available from iNeuro Project^2^.

## A Statement of the Problem: Challenges of Large-Scale Data and Training

Many of the challenges of big data can be linked to the absence of a workforce adequately trained to harness its potential (see *Focus on big data*^6^). Training such a workforce will help alleviate many of the challenges presented. For example, there is an absence of standards and best practices for collecting and curating diverse data sets (Ferguson et al., 2014; Gomez-Marin et al., 2014; Poldrack and Gorgolewski, 2014). Nonetheless, if we train people to work with big data, they will not only have the capacity to handle data within the framework of current standards, but also will have the capacity to create standards for the future so that data can be readily shared, analyzed, and understood at deeper, richer levels.

Another major challenge in large-scale neuroscience data is the integration of both small and large, often heterogeneous data sets into interoperable repositories (Ferguson et al., 2014). Few individuals currently have the skills to accomplish the required integration. Forming a properly trained workforce equal to these tasks is a necessary step to meet this challenge.

Higher education communities have a pressing responsibility to train a workforce that will be able to understand and create standards and ontogenies that will make big data usable. Strong programs are needed to prepare graduates to contribute directly to the conversations that build and revise standards, establish best practices, and integrate across datasets.

## Foundational Questions in Designing Appropriate Educational Programs

iNeuro Project participants considered several organizing questions: (1) What skill sets does a scientist/curator of large-scale neuroscience data need? (2) What degree level(s) should these individuals hold (e.g., 2-year, B.A./B.S., M.A./M.S., M.D., Ph.D.)? (3) What extant programs are providing adequate training? Are there any extant programs that could serve as models? Will we need new academic programs to generate individuals with the desired skills? (4) What is the desired curriculum for programs that train individuals to use large-scale neuroscience data? (5) How can the recommendations for transforming life sciences education provided in *Vision and Change*^1^ inform the curricula for training individuals using large-scale neuroscience data?

### Skill Sets

Neuroinformatics requires scientists with experience in both the “wet” bench sciences and the “dry” computational and data sciences. Few individuals, however, will be able to invest the time necessary to develop complete fluency in both areas. Thus, this workforce requires interdisciplinary training so that students become somewhat conversant in both neuroscience and computation.

The future workforce was envisioned as individuals who could engage effectively with large and diverse data sets. Further, as members of interdisciplinary teams, they would construct valuable repositories and insightfully interrogate data. Accordingly, workshop participants anticipated that this workforce would need specific skills, knowledge, and competencies that fit into four general categories: (a) research; (b) computational; (c) strategic; and (d) relational.

(a) Research Skills: although no individual student will become conversant with all neuroscience methodologies, participants uniformly endorsed training in some neuroscience research skills. Specifically, students should learn at least one suite of experimental techniques as part of their training with a strong emphasis on experimental design and analysis. Insights into how data are collected and characteristically analyzed were seen as important skills for this workforce to have.

(b) Computational Skills: computational and modeling skills such as computing principles, high performance computing techniques, data visualization, programming, database design, web technologies, and data transfer methods were seen as essential. Skills traditionally within the realm of information and library science were also seen as important. These include understanding existing resources, data formats, standards, vocabularies, lexicons, ontologies, semantics, lifecycles, workflows, annotation, metadata, and interoperability. Finally, necessary skills from the quantitative sciences include data analysis, machine learning, programming, coding, scripting, probability, statistics, signal processing, imaging, and standardization of workflows.

(c) Strategic Skills: the proficiencies required by this envisioned workforce go far beyond just “managing” data. They would include curating, translating, revamping, stewarding, hacking, and even advocating for the data. Current and future neuroinformatics practitioners need to be particularly imaginative, nimble, and strategic if they are to engage effectively with large and diverse data sets as well as with other scientists who create and interrogate the data.

(d) Relational Skills: iNeuro Project workshop participants acknowledged that individuals who are poised to make advances in neuroinformatics are those with experiences in communication, collaboration, and ethics. Strong communication skills are critical to build successful teams that function effectively and create collaborations transcending multiple boundaries. Moreover, robust written, oral, and visual communication skills are needed to relate research outcomes to a wide variety of audiences, including scientists, administrators, policy makers, and the general public. Finally, future scientists should balance the demands for shared and open data with relevant ethical considerations including matters of privacy, law, licensing, and attribution responsibilities for various data types.

### Degree Requirements

Rather than a single type of individual, workshop participants foresaw up to three distinct positions in the future workforce of this field: (a) data researcher or computational neuroinformatician/modeler; (b) data steward; and (c) data technician/manager/advocate. A researcher or computational neuroinformatician uses data with more in-depth discipline knowledge and develops new techniques of analyses to advance the field. A steward is a data-curation professional/practitioner who maintains long-term data in a disciplinary repository. A technician/manager/advocate is the acquisitional data manager who likely would be involved throughout projects. The latter two categories of data professionals were viewed as emerging types of positions. They were also seen as essential members of interdisciplinary teams who likely possessed strong training in computer science, data science, and/or library science as well as interest or experience in neuroscience. Moreover, these data professionals play important roles in developing and upholding much-needed standards and best practices for ensuring data consistency, quality, and interoperability (Posey Norris et al., 2015).

Accordingly, different degree levels could accommodate this diverse workforce. Although workshop participants viewed graduate level training as the most appropriate for the positions described above, they also asserted that preparatory work for relevant degree training could be accomplished at the undergraduate or even associates degree level (see “Curricula” Section below).

### Curricula

Foundations at the undergraduate level should include quantitative literacy and hands-on research experience with at least one novel scientific question. iNeuro participants emphasized that undergraduate science curricula should cultivate good data habits where students learn both the value of collecting strong, reproducible data and develop an ethos that explicitly encourages sharing data with others. Finally, programs should encourage undergraduates to develop creative and hacking mindsets that allow them to view challenges as exciting open frontiers to be navigated.

Workshop participants suggested that competitive applicants to masters or doctoral neuroinformatics programs will likely enter with undergraduate degrees in diverse disciplinary foundations. Attractive applicants to Ph.D. programs will have taken such courses as database design, web programming, data structures, script writing, statistics, research methodology and design, ethics, intellectual property, biology, physical science, engineering, psychology, and neuroscience (see also Engert, 2014).

The positions of data steward and technician/manager/advocate will require training at the M.S. degree level or beyond. This training in neuroinformatics would need to address both breadth across transdisciplinary fields, going beyond the bounds of single disciplines to form new holistic viewpoints, and depth within an area of expertise. The curricular requirements should include foundations in hands-on experiences using large datasets with transdisciplinary teams investigating original questions in neuroscience. Such coursework would provide experiences with not only large-scale data, but also with team science and open-ended research challenges. Additionally, participants felt that training in neuroscience, library and information science, computer science, and communication modes (including data visualization) should be included as components of the curricular training for these positions.

The position of computational neuroinformatician, will require a Ph.D. degree, perhaps with augmented emphases as required by big data. A Ph.D. in neuroinformatics would entail breadth across transdisciplinary fields as well as depth within an area of expertise. This training should incorporate substantial original research experiences using large-scale datasets in the setting of collaborative transdisciplinary team environments, investigating original questions in neuroscience. In addition to the curricular elements for M.S. programs, desirable elements of Ph.D. programs will include math (probability, statistics, linear algebra), machine learning, information technology, systems, and networks. Ph.D. training in neuroinformatics should also include both the wet and dry aspects of contemporary information neuroscience. Students should be expected to produce Ph.D. theses directly linking laboratory experimentation (and/or validation) with modeling or informatics using large-scale data sets.

### Extant Programs

Although the needs for training in informatics and data science are now being recognized (Bernstein, 2014), discussions at the iNeuro workshop revealed that most scientists currently engaging in neuroinformatics research developed their skills through *ad hoc* training fueled by a combination of curiosity, necessity, personal motivation, and accessible resources. Further, investigators rarely hire employees that are products of formal training because very few workers in this field are products of a coherent training program or intentional institutional structure in neuroinformatics.

Clearly there are few, if any, extant programs in the United States that could provide the requisite training. There are, however, neuroinformatics programs in Europe that might provide relevant training. A full list of extant training opportunities, both degree granting and short courses, are provided by the INCF^7^. Workshop participants frequently referred to the *Doctoral Training Centre in Neuroinformatics and Computational Neuroscience^8^* at the University of Edinburg as a possible model. A possible undergraduate model is the bachelor’s degree training program in the Department of Biomedical Physics at the University of Warsaw^9^.

### Recommendations

*Vision and Change*^1^ details a series of recommendations that arose from conferences sponsored by NSF and the American Association for the Advancement of Science (AAAS) in 2009 and 2013. Consistent with *Vision and Change*, iNeuro workshop participants endorsed integrating authentic research experiences into educational programs. Ideally, all levels of graduate training would include a team project—a capstone experience that would tackle a real-world, big data neuroscience problem. Clearly, the tools and strategies proposed by *Vision and Change* to initiate and sustain change at the institutional and departmental level will need to be employed to make education in big data neuroinformatics a reality.

## Building iNeuro Programs

**Figure 1:**
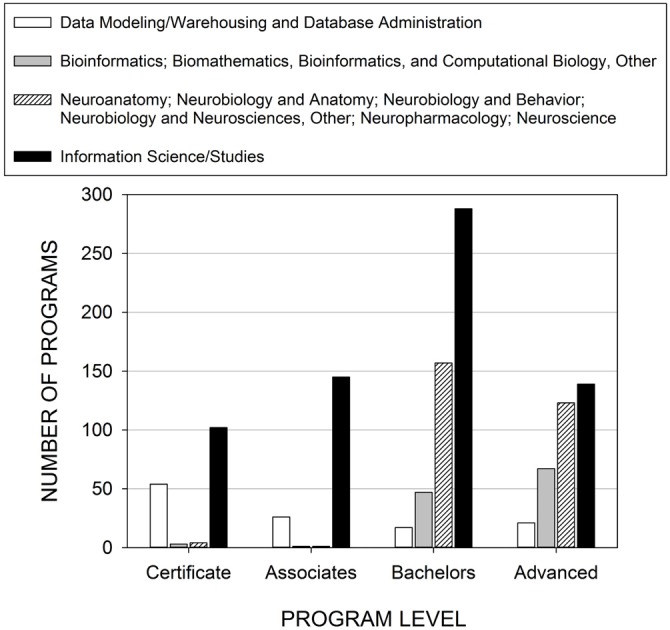
**Number of extant programs in the US offering training in skills needed in big data neuroinformatics.** Only two US institutions offered programs with all four categories. Methodology is similar to that described in Ramos et al. (2011).

Workshop participants suggested that essential curricular elements of neuroinformatics training are currently in place in some institutions, yet they are not coordinated to train a workforce to handle big data in neuroscience. The elements relevant for big data neuroinformatics training are database administration, bioinformatics/computational biology, neuroscience, and information studies. Using data provided by the National Center for Education Statistics (NCES)^10^ and methodology described by Ramos et al. (2011), we noted whether or not each of these elements were present in institutions that offered various degrees (Figure 1; Supplementary Materials). Currently, there are only two US institutions that offer programs with all four of these elements: George Mason University and the University of Nebraska at Omaha.

Neither incentives nor the structure to coalesce these extant program elements into a curriculum addressing the needs of the neuroinformatics workforce are in place, however. If faculty members currently housed in existing departments or programs could be joined around common neuroinformatics goals and provided with appropriate resources, then new degree programs in neuroinformatics could be created largely from existing parts at many institutions. Moreover, many universities have considerable experience building and sustaining interdepartmental graduate programs in related areas such as neuroscience, bioinformatics, life sciences, and applied computation. The lessons learned in creating and sustaining other interdepartmental graduate programs will likely translate readily to launching neuroinformatics programs.

## Conclusions

There are several reasons to initiate big data neuroinformatics programs at this point in time: the public is interested in such efforts, there are expanding global online resources including those provided by the INCF^7^, and job opportunities for such a workforce are already emerging. Further, a consensus on skill sets, curricula, and pedagogical approaches is at hand. iNeuro workshop participants reached a consensus on the skill sets desired and also largely concurred on necessary curricular content appropriate for such programs. Participants endorsed training that was student-centered, hands-on, and included solving real problems in transdisciplinary teams at all degree levels. Workshop participants felt that the curricula discussed above will strengthen the field of neuroscience as a whole (Posey Norris et al., 2015), and that the skills learned are highly transferrable.

Initiating such curricular programs will have costs, however. Proposed training programs have extensive curricular demands that will require resources and faculty development. Adding new courses, much less new programs, will place demands on institutional resources. Start-up funds for initiating a neuroinformatics program might be obtained. The demand for such courses of study exists but its extent is unknown, so the long-term viability of such programs is similarly unknown. An online hub of resources, such as the one constructed by INCF^7^, will be of help in mediating costs.

Possibly, the greatest threat lies with the cost of inaction. Not training people to deal with the imminent onslaught of big data will contribute to the enormous cost of lost opportunities. Large-scale data hold enormous promise, particularly in their potential for synergistic activities, allowing insights and analyses not even envisioned by those who originally collected the data. Further, large-scale data provide unique training opportunities allowing students, including undergraduates, to engage in authentic research and to make real contributions to the knowledge base.

Thus, we need a workforce trained to handle, curate, and utilize large-scale data, otherwise the potential of these data will go unmet. Government, inter-government, and non-governmental agencies may be interested in promoting workshops and other collaborative mechanisms to identify key opportunities for progress in the organization and analysis of large-scale datasets (see *Priorities for Accelerating Neuroscience Research*^3^), and the research and educational communities should press for such collaborative efforts.

## Author Contributions

WG, BL, LL, and RLR: made substantial contributions to the conception and design of the work, drafted and revised the manuscript for intellectual content, gave approval to the final version, and agreed to be accountable for all aspects of the work. RLR: obtained and interpreted the data presented.

## Funding

This work was supported by National Science Foundation Division of Undergraduate Education (NSF DUE) Grant #1441416 “iNeuro: Response to an identified need for a workforce trained to curate and manage large-scale data and databases”.

## Conflict of Interest Statement

The authors declare that the research was conducted in the absence of any commercial or financial relationships that could be construed as a potential conflict of interest.

## Footnotes

^1^http://visionandchange.org/files/2013/11/aaas-VISchange-web1113.pdf

^2^https://mdcune.psych.ucla.edu/ineuro/reports/iNeuro_WorkshopReport_v20160119.pdf

^3^https://www.whitehouse.gov/sites/default/files/microsites/ostp/NSTC/accelerating_neuroscience_research_-_feb_2014.pdf

^4^https://www.humanbrainproject.eu/

^5^https://www.whitehouse.gov/share/brain-initiative

^6^http://www.nature.com/neuro/journal/v17/n11/pdf/nn.3856.pdf

^7^https://www.incf.org/resources/training

^8^http://www.anc.ed.ac.uk/dtc/

^9^http://neuroinformatyka.pl/

^10^https://nces.ed.gov/
